# Brain Functional Network Analysis of Patients with Primary Angle-Closure Glaucoma

**DOI:** 10.1155/2022/2731007

**Published:** 2022-01-06

**Authors:** Dan Liu, Junwei Gao, Tao You, Shenghong Li, Fengqin Cai, Chonggang Pei, Xianjun Zeng

**Affiliations:** ^1^Department of Radiology, Hubei Cancer Hospital, Tongji Medical College, Huazhong University of Science and Technology, Wuhan, 430079 Hubei Province, China; ^2^Department of Radiology, The First Affiliated Hospital of Nanchang University, Nanchang, 330006 Jiangxi Province, China; ^3^Department of Radiology, Chinese PLA General Hospital of Central Theater Command, Wuhan, 430070 Hubei Province, China; ^4^Department of Ophthalmology, The First Affiliated Hospital of Nanchang University, Nanchang, 330006 Jiangxi Province, China

## Abstract

**Objectives:**

Recent resting-state functional magnetic resonance imaging (fMRI) studies have focused on glaucoma-related neuronal degeneration in structural and spontaneous functional brain activity. However, there are limited studies regarding the differences in the topological organization of the functional brain network in patients with glaucoma. In this study, we aimed to assess both potential alterations and the network efficiency in the functional brain networks of patients with primary angle-closure glaucoma (PACG).

**Methods:**

We applied resting-state fMRI data to construct the functional connectivity network of 33 patients with PACG (54.21 ± 7.21 years) and 33 gender- and age-matched healthy controls (52.42 ± 7.80 years). The differences in the global and regional topological brain network properties between the two groups were assessed using graph theoretical analysis. Partial correlations between the altered regional values and clinical parameters were computed for patients with PACG.

**Results:**

No significant differences in global topological measures were identified between the two groups. However, significant regional alterations were identified in the patients with PACG, including differences within visual and nonvisual (somatomotor and cognition-emotion) regions. The normalized clustering coefficient and normalized local efficiency of the right superior parietal gyrus were significantly correlated with the retinal fiber layer thickness (RNFLT) and the vertical cup to disk ratio (V C/D). In addition, the normalized node betweenness of the left middle frontal gyrus (orbital portion) was significantly correlated with the V C/D in the patients with PACG.

**Conclusions:**

Our results suggest that regional inefficiency with decrease and compensatory increase in local functional properties of visual and nonvisual nodes preserved the brain network of the PACG at the global level.

## 1. Introduction

Glaucoma is an age-related, blindness-causing disease characterized by the progressive death of retinal ganglion cells (RGCs) in association with increased intraocular pressure (IOP). Glaucoma may be classified into primary open-angle glaucoma (POAG) and primary angle-closure glaucoma (PACG) according to the anatomy of the anterior chamber angle [1]. However, in China, PACG is still the most common type of glaucoma [2, 3]. Moreover, recent pathological data have demonstrated that neurodegenerative processes in the central nervous system of patients with glaucoma, such as apoptosis and reactive oxygen species production, were similar to the processes identified in Alzheimer's disease (AD) [4, 5], which suggests that glaucoma is a neurodegenerative disease. Therefore, potential alterations in the central nervous system of patients with PACG should receive greater emphasis.

Structural neuroimaging, including voxel-based morphometry (VBM) and diffusion tensor imaging (DTI), indicates an atrophic optic nerve (ON) and lateral geniculate body (LGN), damaged optic radiation (OR), and a thinning visual cortex in glaucoma patients [6–9]. Functional magnetic resonance imaging (fMRI) has demonstrated several forms of visual task-related deactivation in glaucoma [10], including decreased visual functional connectivity (FC) [11], decreased and/or increased amplitude of low-frequency fluctuations (ALFF) in the cuneus and occipital lobe/inferior temporal gyrus [12–14], and decreased regional homogeneity (ReHo) in the calcarine area [15]. In addition, structural and functional studies have indicated alterations in the extravisual system, such as the hippocampus [16], anterior thalamic radiation, superior longitudinal fascicle [17], and executive network [18]. However, these mentioned studies have primarily focused on regional alterations in structures and/or functions of the brain [6–18] and ignored alterations in the functional network architecture and information transfer which may indicate the changes of visual and nonvisual pathway in patients with PACG. Thus, there might need some other researches for a more comprehensive understanding of visual and extravisual processes changes of patients with PACG.

Recent graph theoretical developments have become increasingly popular for describing the structural and functional network maps of the human brain. The human brain is a complex and interconnected network characterized by efficient, coordinated, and integrated neuronal activities, with nodes that represent neurons and edges that represent internodal neural connections [19, 20]. The topological features have been well defined, such as small-worldness [21–23], global and local efficiency [24], and highly connected hubs [25], which contain node degree, node betweenness, and so on. The brain network features have been characterized in AD [26, 27], schizophrenia [28], multiple sclerosis [29], and other conditions. Moreover, several analyses have indicated that patients with PACG exhibit signs of changes in brain network properties that were identified in previous resting-state fMRI studies. For example, Cai et al. identified altered features of degree centrality (DC) at voxel levels in patients with PACG [30]. Based on the different patterns of functional connectivity between PACG and POAG patients, it is reasonable to question whether the topological properties of the whole brain network are also altered in patients with PACG.

We hypothesized that patients with PACG would exhibit significantly altered global network topological properties, such as small-worldness, global efficiency, or normalized clustering coefficients. The areas considered in the assessment of this hypothesis included 116 regions defined by the Automated Anatomical Labeling (AAL) atlas. The alterations in the brain network pattern were correlated with clinical parameters to investigate the potential relationship between network topological properties and clinical parameters. It may provide for PACG a unique opportunity to understand how the brain network topological organization changes in response to ocular hypertension and secondary neural degeneration.

## 2. Materials and Methods

### 2.1. Subjects

Thirty-three patients with PACG (13 M/20 F, mean age 54.21 ± 7.21 years) were recruited from the Department of Ophthalmology of the First Affiliated Hospital of Nanchang University. Thirty-three age- and gender-matched healthy controls were recruited through local advertisements. All subjects were right-handed and participated in this study after providing written informed consent. The study was conducted in accordance with the Declaration of Helsinki. The study protocol was approved by the Institutional Review Board of the First Affiliated Hospital of Nanchang University. All patients with PACG underwent a detailed ophthalmological examination, including gonioscopy, IOP assessment, fundoscopy, and standard automated perimetry.

The inclusion criteria for the patients with PACG were as follows: (1) narrow anterior chamber angle in one or both eyes, (2) characteristic optic disc damage (optic disc cupping or thinning), and (3) typical glaucomatous visual field loss (tubular vision or central island). The exclusion criteria for the patients were as follows: (1) secondary glaucoma or other ocular disorders; (2) neural-associated diseases, chronic pain, hypertension, diabetes, or a history of brain surgery; or (3) inability to attend MRI scanning because of metal implantation or a history of claustrophobia or other psychological disorders.

### 2.2. MRI Data Acquisition

All MRI data were collected on a 3.0 T (Trio Tim, Siemens, Erlangen, Germany) scanner using an 8-channel phased-array head coil. During the MRI scanning, each participant laid in the supine position, with the head in a neutral position and fixed comfortably by a belt and foam pads. All participants were required to relax, think of nothing in particular, remain awake, and keep their eyes closed. (1) The resting-state functional MRI (fMRI) data were acquired using a gradient-echo echo-planer imaging (EPI) sequence with the following parameters: repetition time (TR) = 2,000 ms, echo time (TE) = 40 ms, flip angle = 90°, slice thickness/gap = 4.0/1 mm, field of view (FOV) = 240 mm × 240 mm, in − plane resolution = 64 × 64, 30 axial slices that covered the whole brain, and 240 volumes acquired in 8 min. (2) The high-resolution brain structural images of each subject were acquired using a T1-weighted 3D MP-RAGE sequence as follows: TR = 1,900 ms, TE = 2.26 ms, flip angle = 9°, matrix = 256 × 256, FOV = 240 mm × 240 mm, thickness = 1.0 mm, and 176 sagittal slices.

### 2.3. MRI Data Preprocessing

The Data Processing Assistant for Resting-State fMRI (DPARSF, http://www.restfmri.net), which is based on Statistical Parametric Mapping (SPM8) (http://www.fil.ion.ucl.ac.uk) and the Resting-State Data Analysis Toolkit (REST, http://www.restfmri.net), was used for all fMRI data preprocessing. The first ten volumes of the resting-state fMRI data were discarded for each participant to remove potential effects of scanner instability and adjust for the surroundings. The remaining 230 volumes were corrected for slice timing. The resultant images were realigned to correct for small movements. In addition, participants with head displacements that exceeded 3 mm in the cardinal directions (*x*, *y*, *z*) and 3° of rotation (*x*, *y*, *z*) were excluded. The T1-weighted structural images used for segmentation were coregistered to the corrected functional images for each individual and segmented into GM, WM, and CSF [31]. Based on the transformation matrix, the functional images were spatially normalized to standard Montreal Neurological Institute (MNI) space and subjected to nonlinear modulation to compensate for spatial normalization effects. All the standard MNI space functional images were smoothed with a 6 mm full-width at half maximum Gaussian kernel [32].

To further remove the effects of nuisance covariates, the signals from the white matter, cerebrospinal fluid, global signals from the entire brain, and six head realignment parameters used for rigid body head motion correction were removed from the data through linear regression [33]. Temporal filtering (0.01-0.1 Hz) of the time series was subsequently performed to remove very low-frequency drifts and physiological high-frequency noise.

### 2.4. Network Construction

A brain network may be considered as a graph, G (V, E), composed of vertex (V) and edge (E). The 116 brain regions from the AAL template [34] are the vertexes in the graph, and the Pearson coefficients of each region pair (e.g., the *i*-th region and the *j*-th region) represent the edges between two vertexes (*wij*). First, the time series of each node were extracted by averaging the time series of all voxels in the same nodal areas. Then, the Pearson correlation coefficients between the time series of each possible node pair were calculated. Thus, a functional correlation matrix was generated for each subject [35]. To improve the normality of the correlation coefficient, Fisher's *r*-to-*z* transformation was applied to the correlation matrices [36].

A previous study suggested that the brain network of each normal subject differs in both the number of significant edges and their weights [37]. Therefore, the threshold method was used to determine the available edges in the matrix and binarize the entire matrix. As there is no consensus in regard to the setting of functional-network-threshold to date, we employed a wide range of sparsity thresholds (range from 0.1 to 0.6, with step = 0.01) to analyse alterations in the topological properties of the brain network in the two groups. The minimum network sparseness can ensure that all nodes in the network are connected, and the maximum network sparseness can maintain the small-world attributes of the network.

### 2.5. Network Analysis

The network analysis toolbox, GRETNA (Version 1.0, http://www.nitrc.Org/projects/gretna/), running on a matrix laboratory platform (MATLAB R2010a; Natick, MA, USA), was used to estimate the topological properties of the networks [38]. Five small-world parameters and two efficiency parameters were used to assess the global topological organization of the functional brain networks in the PACG and HC groups. Four regional parameters were computed to characterize the properties of the 116 brain regions.  Table 1 provides a brief description of the network properties, and Supplementary Materials 1 provides the definitions and calculations.

### 2.6. Statistical Analysis

Differences in the global (*C*_*p*_, *L*_*p*_, *λ*, *γ*, *σ*, *E*_glob_, and *E*_loc_) and regional (normalized Deg(*i*), NB(*i*), *E*_loc_(*i*), and *γ*(*i*)) topological parameters between the PACG and HC groups were examined in MATLAB. The network measure data basically complied with the normality which were tested by using SPSS to apply the Kolmogorov-Smirnov test. Regional differences were also identified at a minimum sparsity of 0.32, which maintains the integrity of functional networks, whereas *D*_max_ was set at 0.42 to ensure the small-worldness of the two groups. As a result, differences in the global network properties and regional network properties between the PACG and HC groups were compared using a two-sample *t*-test (false discovery rate (FDR), *p* < 0.05) over the small-world network sparsity threshold (from 0.32 to 0.42). We ultimately correlated the regional values of the altered regions in the patients with PACG with the RNFL, V C/D, and IOP at a minimum sparsity of 0.32. SPSS v.17.0 was used for this analysis.

## 3. Results

### 3.1. Participant Characteristics

The demographic information of the patients with PACG and controls is listed in Table 2. There was no significant difference in age and gender between the 33 PACG group and 33 HC group (*p* < 0.05).

### 3.2. Global Network Property Changes

**Figure 1:**
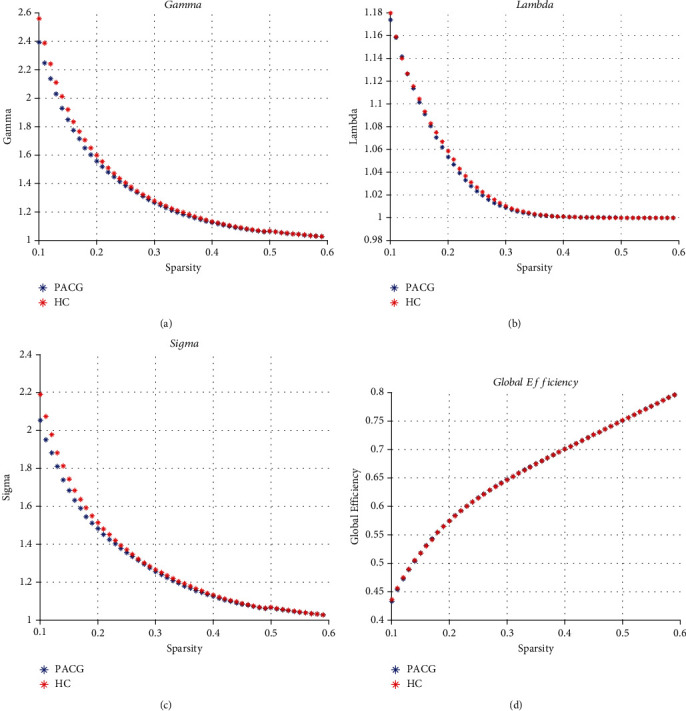
Changes in global network property measures as a function of network sparsity (from 0.1 to 0.6). The normalized clustering coefficient *γ* (a), normalized characteristic path length *λ* (b), small-world index *σ* (c), and global efficiency (d) of both the PACG and HC networks. Both networks follow a small-world organization.

The global functional network properties and two efficiency parameters in the two groups are presented in Figure 1. The networks of both the PACG and HC groups exhibited small-world characteristics (*γ* > 1, *λ* ≈ 1, or *σ* > 1) at all network sparsities (0.1-0.6).

**Figure 2:**
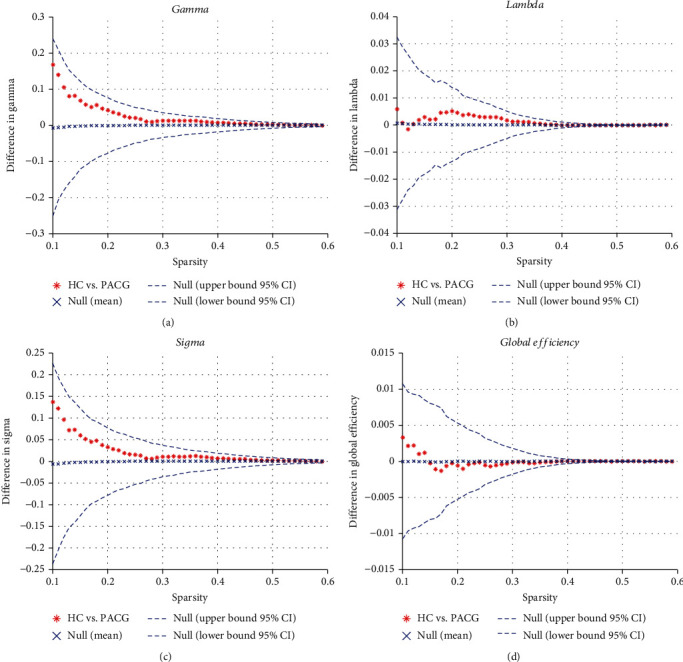
Between-group differences in global network measures as a function of small-world network sparsity (from 0.32 to 0.42). The 95% confidence intervals and between-group differences for the normalized clustering coefficient *γ* (a), normalized path length *λ* (b), small-world index *σ* (c), and global efficiency (d). Compared with the HC network, the global network normalized clustering coefficient and small-world index in the PACG network were slightly different across the small-world network sparsity range.

Over the sparsity range of 0.32 to 0.42, the functional brain networks in the PACG group exhibited a slightly lower clustering coefficient (*p* = 0.904 ~ 0.999) and normalized clustering coefficient (*p* = 0.283 ~ 0.453), similar characteristic path length (*p* = 0.545 ~ 0.996) and normalized characteristic path length (*p* = 0.463 ~ 0.993), and slightly lower small-worldness (*p* = 0.322 ~ 0.548) compared with the HCs. No statistically significant differences were identified in these regards (*p* > 0.05) (Figure 2). Moreover, the PACG group exhibited a similar mean local efficiency and whole brain global efficiency compared with the HC group.

### 3.3. Regional Network Property Changes

**Figure 3:**
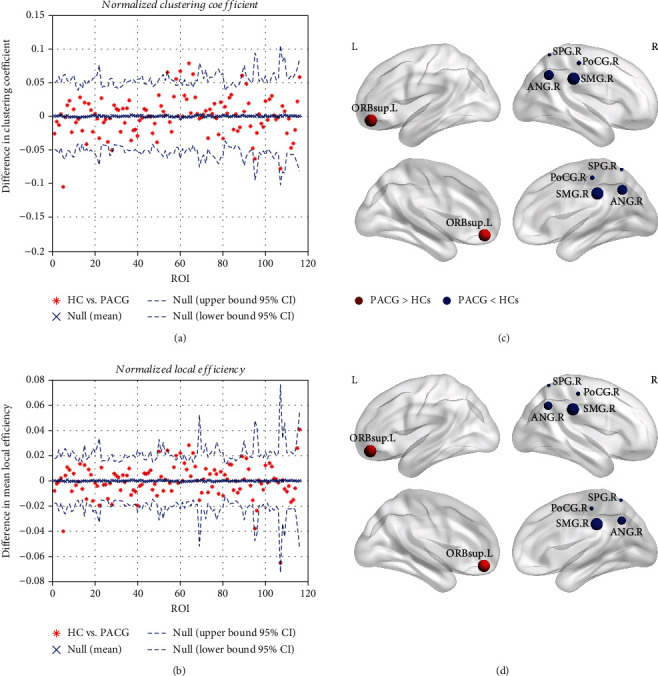
Regional alterations in normalized clustering coefficiencies and normalized local efficiencies in functional brain networks at a minimum sparsity of 0.32. Statistical graphs of each node are presented on the left (a, b), and a three-dimensional view of the corresponding significant nodes are shown on the right (c, d). The significantly increased regional nodal parameters (normalized clustering coefficient and local efficiency) of patients with PACG are indicated by the red spheres, and significantly increased regional nodal parameters of the HC group are indicated by the blue spheres. The sphere sizes represent the significance values (*p* < 0.05, FDR correction); larger spheres indicate greater significance and smaller *p* values.

The differences in the regional normalized clustering coefficients and local efficiencies between the two groups are presented in Figure 3 at a sparsity threshold of 0.32. Compared with the HC group, a decreased normalized clustering coefficient and local efficiency were identified in the dorsal visual pathway brain regions (right superior parietal gyrus and right angular gyrus) and nonvisual pathway brain regions (right postcentral gyrus and right supramarginal gyrus) in the PACG group. In contrast, these metrics were increased in the left superior frontal gyrus (orbital portion).

**Figure 4:**
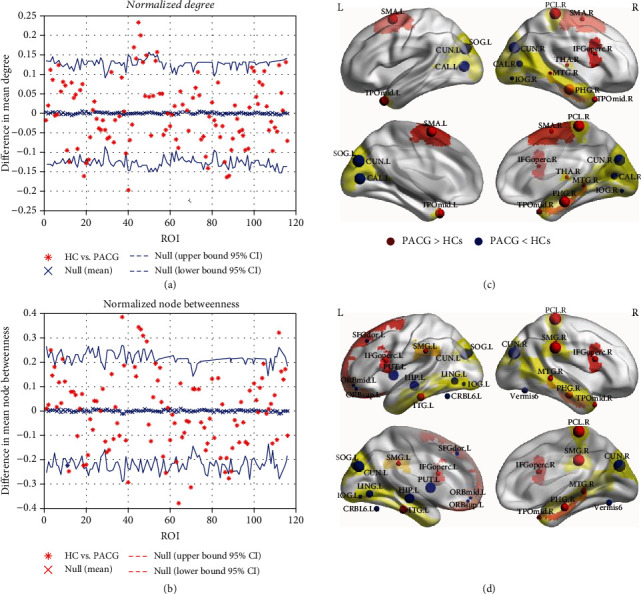
Regional alterations in normalized node degree and normalized node betweenness in functional brain networks at the minimum sparsity of 0.32. Graphs of each node are presented on the left (a, b), and a three-dimensional view of the corresponding significant brain regions are shown on the right (c, d). The significantly increased regional nodal parameters (normalized node degree and normalized node betweenness) of the patients with PACG are indicated by the red spheres, and the significantly increased regional nodal parameters of the HC group are indicated by the blue spheres. The sphere size represents significance (*p* < 0.05); a larger sphere size indicates greater significance and a smaller *p* value.

The difference in the regional normalized node degree between the two groups is presented in Figure 4 at a sparsity threshold of 0.32. The PACG patients exhibited a decreased normalized degree in the primary and secondary visual cortices (bilateral calcarine, left superior occipital gyrus, right inferior occipital gyrus, and bilateral cuneus). An increased normalized degree was identified in the dorsal visual pathway brain regions (right thalamus and bilateral middle temporal gyrus), as well as nonvisual pathway brain regions (bilateral supplementary motor area, right parahippocampal, right paracentral lobule, and right inferior frontal (opercular portion)).

Figure 4 and Table 3 indicate differences in the normalized node betweenness between the PACG and HC groups. The normalized node betweenness in the dorsal visual pathway brain regions (left superior occipital gyrus, left inferior occipital gyrus, bilateral cuneus, and left lingual gyrus) was decreased in the patients with PACG. A decrease was also identified in the nonvisual regions, including the left superior frontal gyrus, left superior frontal gyrus (orbital portion), left middle frontal gyrus (orbital portion), left hippocampus, left putamen, left cerebellum, and vermis. Furthermore, the patients with PACG exhibited significant increases in the normalized node betweenness in the dorsal/ventral visual pathway (right middle temporal gyrus, right middle temporal pole, and left inferior temporal gyrus) as well as in nonvisual areas (right parahippocampal, right paracentral lobule, bilateral inferior frontal gyrus (opercular portion), and bilateral supramarginal gyrus) (*p* < 0.05).

### 3.4. Correlation between Altered Regional Values in Patients with PACG and Clinical Parameters

**Figure 5:**
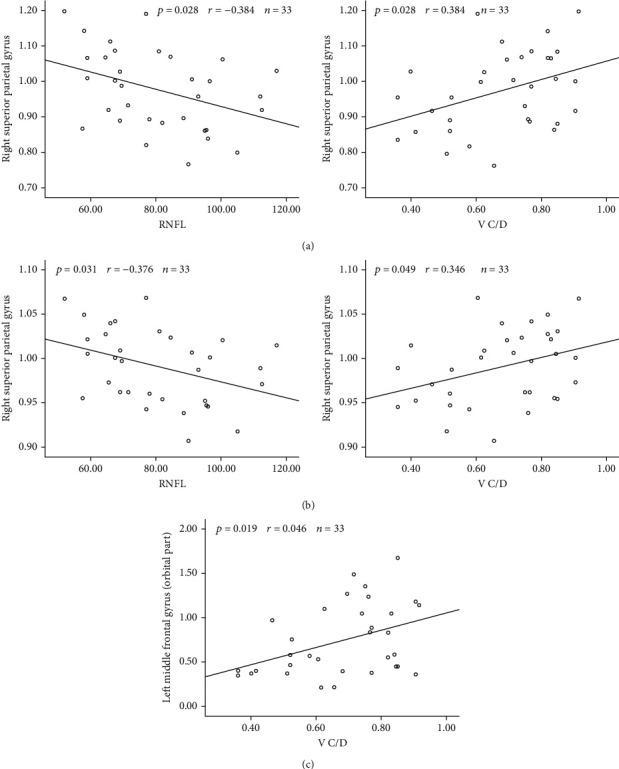
Correlation between altered regional values in patients with PACG and clinical parameters. The right superior parietal gyrus of the normalized clustering coefficient is correlated with the RNFL and V C/D (a), the right superior parietal gyrus normalized local efficiency is correlated with the RNFL and V C/D (b), and the left middle frontal gyrus (orbital portion) normalized node betweenness is correlated with the V C/D (c).

We correlated the clinical parameters with the regional values of brain areas that exhibited alterations in the patients with PACG (Figure 5). We determined that the normalized clustering coefficient of the right superior parietal gyrus was negatively correlated with the RNFL (*r* = −0.384, *p* = 0.028) and positively correlated with the V C/D (*r* = 0.384, *p* = 0.028). Similarly, the normalized local efficiency of the right superior parietal gyrus was negatively correlated with the RNFL (*r* = −0.376, *p* = 0.031) and positively correlated with the V C/D (*r* = 0.346, *p* = 0.049). In addition, the normalized node betweenness of the left middle frontal gyrus (orbital region) was positively correlated with the V C/D (*r* = 0.406, *p* = 0.019).

## 4. Discussion

The present study investigated the topological properties of the functional brain networks of patients with PACG at both global and regional levels. Our main findings are as follows: (1) an economical and stabilized small-world property is identified in the whole brain functional networks of patients with PACG; (2) patients with PACG exhibit significantly altered regional characteristics (normalized degree, node betweenness, local efficiency, and clustering coefficient) in several regions, including visual and nonvisual pathways; and (3) in patients with PACG, the normalized clustering coefficient and local efficiency of the right superior parietal gyrus were significantly correlated with the RNFL and V C/D, whereas the normalized node betweenness of the left middle frontal gyrus (orbital portion) was significantly correlated with the V C/D.

### 4.1. Economical and Stabilized Global Network Properties

The small-world network is considered to be well balanced with local brain specialization and global integration. Our study indicated that the networks of the two groups had economic small-world characteristics (*γ* > 1, *λ* ≈ 1, or *σ* > 1).

In our study, the specific maximum and minimum sparsity thresholds were set to range from 0.32 to 0.42 to avoid the existence of isolated nodes and ensure small-worldness in both group networks. We determined that the global features of the brain network did not exhibit significant changes in patients with PACG above the sparsity threshold. Recently, Wang et al. [42] employed graph theoretical analysis and reported no statistical changes in global brain properties in POAG patients, which suggests that the efficiency of functional brain network information transmission was conserved. Consistent with this previous study, our results indicated that the brain network was homogeneous [ [43]] in glaucoma patients. However, the clustering coefficient (*λ*) and small-worldness (*σ*) were slightly decreased (*p* > 0.05), which indicates that the global brain network characteristics of patients with PACG tend to decrease. This finding suggests that the balance of local specialization and global integration in patients with PACG was disrupted, which caused the brain network in patients with PACG to tend towards a lower efficiency random network.

### 4.2. Alteration of Regional Network Properties

The analysis of regional network properties in patients with PACG indicated differences in the normalized node degree and normalized node betweenness in both visual and nonvisual pathways. The node degree and node betweenness are well established to indicate the significance of a node and represent the number of connections with and degree of information processing to all other nodes in a network, respectively [30].

Over the sparsity range of 0.32-0.42, our results indicated a significantly decreased normalized degree in the primary and secondary visual cortices (bilateral calcarine, left superior occipital gyrus, right inferior occipital gyrus, and bilateral cuneus) in patients with PACG, as well as significantly decreased normalized node betweenness in the dorsal visual pathway regions (extending from the left superior occipital gyrus to the left inferior occipital gyrus, bilateral cuneus, and left lingual gyrus). The primary visual cortex (calcarine, occipital lobe, and cuneus) receives direct visual information from the ON and LGN. The primary cortex sends most of its projections to the secondary visual cortex (occipital lobe). Notably, reduced visual stimulation in the visual cortices may lead to a loss of projections from these areas in glaucoma, an effect that leads to impairments in the primary and secondary visual cortices [44]. As similar with previous researches, these impairments include structural damage and functional impairment in the calcarine area, cuneus [ [45, 46]], and occipital lobe [ [45, 47]]. Similarly, the DC is decreased in the superior/inferior occipital and lingual gyri. As a result, the integrity of brain regions in the dorsal visual pathway, which plays an important role in visuospatial function, is further affected. Previous studies have also reported structural changes in the occipital lobe and altered cortical thickness in the cuneus and lingual gyrus [30], which strongly affect visuospatial and motion processing in glaucoma patients. Consistent with these findings, the observed decrease in the normalized node degree and normalized node betweenness indicates a lower efficiency of functional communication through these regions. This negative effect particularly affects visual projection and processing, thereby contributing to an increasing body of evidence of impaired spatial orientation in patients with PACG.

Moreover, the brain regions of the dorsal visual pathway (right thalamus and bilateral middle temporal gyrus) exhibited an increase in the normalized degree, and the dorsal/ventral visual pathway brain regions (right middle temporal gyrus, right middle temporal pole, and left inferior temporal gyrus) exhibited increased normalized node betweenness. Areas that extend from the primary visual cortex to the temporal lobes (middle and right inferior temporal gyrus) may be associated with visual memory or episodic memory imagery [48]. The primary visual cortex and the inferior temporal gyrus are thought to play a role in connecting visual, auditory, and tactile stimulation [49]. Analogous results have also been reported in terms of the synchronization of spontaneous activity [50], and functional integration in the temporal lobe and middle/inferior temporal gyrus exhibits compensatory increases in glaucoma. This effect may be related to neuroplasticity and compensatory mechanisms. Specifically, glaucomatous visual loss may lead to a loss of inhibition inputs of the primary visual regions, following which the dorsal/ventral visual regions are further compensatory increased [51, 52]. Our preliminary study of increased visual cortices in postoperative patients with PACG supports this interpretation [53]. Consistent with these results, our finding of an increased normalized node degree and normalized node betweenness in visual cortices may suggest a compensatory response of visual memory and the integration of visual, auditory, and tactile inputs.

Moreover, we identified a significantly altered normalized node degree and normalized node betweenness in nonvisual cortices, including (i) cognition-emotion processing brain regions, such as the left superior frontal gyrus, left superior/middle frontal gyrus (orbital portion), bilateral inferior frontal lobe (opercular portion), left hippocampus, right parahippocampus, left cerebellum and vermis and (ii) the somatomotor cortex (bilateral supplementary motor area, right paracentral lobule, and bilateral supramarginal gyrus along with left putamen). A recent study suggested that glaucoma patients, particularly patients with PACG, are at a higher risk of anxiety and depression [ [54]]. Moreover, the cerebellum and hippocampus [55], which play an important role in cognition-emotion processing, as well as the frontal lobe (orbital portion) and inferior frontal gyrus (opercular portion) [56] are functionally and structurally disrupted in this condition. In addition, the functional connectivity of somatomotor regions is altered in patients with glaucoma. Consistent with these findings, an altered normalized node degree and normalized node betweenness in nonvisual cortices suggest that the efficiency of cognition-emotion and somatomotor processing is impaired in patients with PACG. This result implies that glaucoma is a widespread brain disorder.

Interestingly, the identified alterations in the normalized clustering coefficients of the dorsal visual pathway and the somatomotor and cognition-emotion regions were similar to the alterations in the normalized local efficiency in patients with PACG. These results are consistent with the hypothesis that the normalized clustering coefficient is a good approximation of efficiency [57]. A substantial proportion of fMRI studies have reported that glaucoma is not simply an ophthalmic disease but rather a more complex, widespread disorder that through visual and nonvisual cortices [50, 58, 59]. Therefore, with the changed visual and nonvisual pathways of normalized clustering coefficient and normalized local efficiency, our results also provided a growing evidence of the conception that glaucoma was not only an ocular disease but also a more widespread brain syndrome.

In our study, the normalized clustering coefficient and normalized local efficiency of the right superior parietal gyrus were negatively correlated with the RNFL and positively correlated with the V C/D. In addition, the decrease in the node betweenness of the left middle frontal gyrus (orbital portion) was positively correlated with the V C/D. The RNFL and V C/D are considered to be important measures of glaucoma severity; i.e., more severe glaucoma is associated with a thicker RNFL and higher V C/D. As a result, these significant correlations indicate that the altered regional properties of the right superior parietal gyrus and left middle frontal gyrus (orbital portion) are correlated with the PACG severity.

## 5. Limitations

Several limitations of this study should be addressed. First, being identified from functional brain network connectivity metrics, the structural brain network is not yet described or characterized as a within-group description; thus, the correlation between structural and functional network should be considered in the future. Second, the PACG sample size was relatively small, and a large longitudinal sample is required for further analysis. Third, our study adopted a binary, undirected network analysis. To address the connectivity strength, a weighted network with a sparsity threshold or an effective connectivity network should also be considered in future analyses. The severity of the damage to the eyes caused by PACG is variable. Moreover, it is doubtful that alterations in brain network properties will be similar between different disease stages and/or severities.

## 6. Conclusions

In conclusion, we investigated the intrinsic functional brain network in patients with PACG using graph theoretical analysis. No significant differences were identified in the global topological measures of patients with PACG; however, significant alterations were identified in the local properties of visual, somatomotor, and cognition-emotional regions. Taken together, our findings indicate that reduced efficiencies in visual and nonvisual processing regions are accompanied by compensatory increases in the local functional properties of visual and nonvisual nodes, which thereby preserve the global network stabilization in patients with PACG. Our framework may help to promote further studies towards a better understanding of the neurological basis of PACG.

## Figures and Tables

**Table 1:** Brief description of network properties in this study.

Global network properties.	
Clustering coefficient *C*_*p*_	*C*_*i*_ is formed by a direct connection to the nearest neighbours of the node *i*, which measures the segregation of the network [39].
	The *C*_*p*_ of a network is the average of *C*_*i*_ over all nodes [39].
Characteristic path length *L*_*p*_	*L*_*p*_ is the average of all of the shortest path lengths between two pairs of nodes, which may be regarded as a measurement of network integration [40].
Normalized clustering coefficient *γ*	*γ* is the average of *C*_*p*_, which is further normalized by comparing to 1000 matched random networks [40].
Normalized characteristic path length *λ*	*λ* is the average of *L*_*p*_, which is further normalized by comparing to 1000 matched random networks [40].
Small-worldness *σ*	*σ* is the ratio of *γ* to *λ*. The network topology may be considered to correspond to a “small world” if *γ* > 1, *λ* ≈ 1, or if *σ* > 1 [39].
Global efficiency *E*_glob_	*E*_glob_ and *E*_loc_ are the biologically sensible topological features of the brain networks, which describe the global and local efficiency of information transmission of the network [24].
Local efficiency *E*_loc_	

Regional network properties	
Normalized degree, Deg(*i*)	Normalized Deg(*i*) is the mean Deg(*i*) of 1000 matched random networks. Deg(*i*) is the number of connections of node *i* that connect it to the rest of the network.
Normalized node betweenness, NB(*i*)	Normalized NB(*i*) is the mean NB(*i*) of 1000 matched random networks. NB(*i*) may be used to compute the effects node *i* that has over the stream of information between all other nodes in the network.
Normalized local efficiency *E*_loc_(*i*)	Normalized *E*_loc_(*i*) is the mean *E*_loc_(*i*) of 1000 matched random networks. *E*_loc_(*i*) is defined as the inverse of the average shortest path length between node *i* and all other nodes. This variable is a measure of information transfer from node *i* to all other nodes of a network [41].
Normalized clustering coefficient *γ*(*i*)	*γ*(*i*) is the mean *γ* of 1000 matched random networks.

**Table 2:** Demographics and clinical characteristics of patients with PACG and controls.

	PACG	HCs	*p* value
Age (years old)	54.21 ± 7.21	52.42 ± 7.80	0.337
Sex (M/F)	13/20	13/20	0.999
Handedness	33R	33R	0.999
RNFL (*μ*m)	80.83 ± 17.69		
Vision	0.46 ± 0.34		
V C/D	0.68 ± 0.17		
IOP (mmHg)	26.86 ± 9.44		

RNFL: retina nerve fiber layer; RNFLT: retina nerve fiber layer thickness; V C/D: vertical cup to disc ratio; IOP: intraocular pressure; M: male; F: female; R: right.

**Table 3:** Between-group comparisons of PACG vs. HCs in regional network properties at a minimum sparsity of 0.32.

Normalized node betweenness		Normalized degree	
Regions	*p* value	Regions	*p* value
PACG > HCs		PACG > HCs	
L. inferior frontal gyrus (opercular portion), IFGoperc.L	0.040	R. inferior frontal gyrus (opercular portion), IFGoperc.R	0.042
R. inferior frontal gyrus (opercular portion), IFGoperc.R	0.024	L. supplementary motor area, SMA.L	0.013
R. parahippocampal gyrus, PHG.R	0.013	R. supplementary motor area, SMA.R	0.041
L. supramarginal gyrus, SMG.L	0.036	R. parahippocampal gyrus, PHG.R	0.012
R. supramarginal gyrus, SMG.R	0.003	R. paracentral lobule, PCL.R	0.002
R. paracentral lobule, PCL.R	≤0.001	R. thalamus, THA.R	0.044
R. middle temporal gyrus, MTG.R	0.025	R. middle temporal gyrus, MTG.R	0.047
R. temporal pole:middle temporal gyrus, TPOmid.R	0.040	L. temporal pole:middle temporal gyrus, TPOmid.L	0.018
L. inferior temporal gyrus, ITG.L	0.012	R. temporal pole:middle temporal gyrus, TPOmid.R	0.038
PACG < HCs		PACG < HCs	
L. dorsal lateral superior frontal gyrus, SFGdor.	0.043	L. calcarine, CAL.L	0.007
L. superior frontal gyrus (orbital portion), ORBsup.L	0.049	R. calcarine, CAL.R	0.032
L. middle frontal gyrus (orbital portion), ORBmid.L	0.049	L. cuneus, CUN.L	≤0.001
L. hippocampus, HIP.L	0.009	R. cuneus, CUN.R	0.004
L. cuneus, CUN.L	0.004	L. superior occipital gyrus, SOG.L	0.040
R. cuneus, CUN.R	≤0.001	R. inferior occipital gyrus, IOG.R	0.042
L. lingual gyrus, LING.L	0.014		
L. superior occipital gyrus, SOG.L	0.012		
L. inferior occipital gyrus, IOG.L	0.047		
L. putamen, PUT.L	0.007		
L. cerebellum 6, CRBL6.L	0.026		
Vermis 6, Vermis6	0.015		

L: left; R: right; PACG > HCs: increased regional network properties in PACG compared with HCs; PACG < HCs: decreased regional network properties in PACG compared with HCs.

## Data Availability

The raw/processed data required to reproduce these findings cannot be shared at this time as the data also forms part of an ongoing study.

## Supplementary Materials

Supplementary Materials 1: the definitions and calculations of network measures.
