# Supplementary material for: Brain Functional Network Analysis of Patients with Primary Angle-Closure Glaucoma
Source: Dis Markers. 2022 Jan 6;2022:2731007. doi: 10.1155/2022/2731007 (PMC8758296; doi:10.1155/2022/2731007)
Supplement: Supplementary Materials — 1: the definitions and calculations of network measures. [file 2731007.f1.docx]

| Supplementary Materials 1. Brief calculation of network properties in this study. | | |
| --- | --- | --- |
| Global network properties | | |
| Clustering Coefficient *Cp* | $C_{i}=\frac{1}{N(N_{G_{i}}-1)}\sum_{j,k\in N_{G_{i}}} 1/L_{jk}$  $C_{p}=\frac{1}{N}\sum_{i\in G} C_{i}$ | *Gi* is the number of edges connected to node *i*. *Ljk* is the shortest path length that links *j* and k in graph *G*. |
| Characteristic Path Length *Lp* | $L_{p}=\frac{1}{N(N-1)}\sum_{i\neq j\in G} L_{ij}$ | *Lij* is the shortest path length that links *i* and *j* in graph *G*. |
| Normalized Clustering Coefficient *γ* | $\gamma={C_{p}}/{C_{rand}}$ | Here, rand is the 1000 matched random networks that preserve the same number of nodes, edges, and degree distribution as the real networks. |
| Normalized Characteristic Path Length *λ* | $\lambda={L_{p}}/{L_{rand}}$ | - |
| Small-worldness *σ* | $\sigma=\gamma/\lambda$ | - |
| Global Efficiency *Eglob* | $E_{glob}(G)=\frac{1}{N(N-1)}\sum_{i\neq j\in G} \frac{1}{d_{ij}}$ | Here, *dij* is the shortest path length between nodes *i* and *j*. |
| Local Efficiency *Eloc* | $E_{loc}(G) =\frac{1}{N}\sum_{i\in G} E_{glob}(G_{i})$ | *Eglob(Gi)* is the global efficiency of *Gi*. |
| Regional network properties | | |
| Normalized Node Betweenness *NB(i)* | $NB(i)=\sum_{i\neq j\neq k\in G} \frac{\sigma_{j,k}(i)}{\sigma_{j,k}}$ | *σj,k* is the number of shortest paths between nodes *j* and *k*, and *σj,k(i)* is the number of shortest paths between nodes *j* and *m* that pass through node *i*. |
